# Evaluation of a method to measure the ratio of pelvic limb to thoracic limb girth in dogs

**DOI:** 10.18849/ve.v8i4.667

**Published:** 2023-10-25

**Authors:** Elisabeth A. Fox+, Kirsty E. Oliver+, Matthew W. Brunke+

**Keywords:** LIMB GIRTH, LIMB MEASUREMENTS, THORACIC, PELVIC, LIMB RATIO, GULLICK, MUSCLE MASS, DOGS

## Abstract

Objective: To determine if a landmark-incorporated limb girth measurement in sound dogs, utilising the greater trochanter and the acromion process, would be both reliable and have a consistent ratio between pelvic limb (PL) and thoracic limb (TL) muscle measurements.

Background: To establish a reliable and reproducible reference range for sound dogs. This reference range may help clinicians further evaluate dogs during lameness and musculoskeletal examinations.

Evidentiary value: Prospective study of 115 sound dogs measured by a single observer.

Methods: Examinations were performed by one observer, using a Gullick II tape measure for PL to TL ratio (PL:TL) measurements for 115 dogs. Bodyweight, breed, body condition score (BCS), and sex were recorded. Each limb was measured three times. The average PL:TL per dog was calculated. Further statistical analysis was used to calculate intra-observer variance and the correlation of limb girth to body weight, BCS, and sex, with significance set at P < 0.05.

Results: The average PL:TL of the sample was 1.515 ± 0.049. Fifty-two dogs of the 115 cases (45%) had a PL:TL ratio of 1.500. PL:TL measurements were not related to dog weight, BCS, or sex. The intra-class correlation was reported to be 0.99.

Conclusion: Our study suggests that a landmark-incorporated measurement in a weight-bearing position can be reproducible. Further investigation is required to determine if this measurement can be reproducible between multiple observers.

Application: A landmark-incorporated limb girth measurement may guide clinicians in case progression and help pinpoint subclinical musculoskeletal disease in dogs.

## Introduction

Limb girth measurements are anthropometric measurements that have been specifically used in human sports medicine and physical therapy to determine muscle mass, which is an indirect method of evaluating muscle strength (Chen et al., 2013). Thigh circumference has been used in human medicine to evaluate strength (Doxey, 1987) and progress of postoperative recovery (Järvelä et al., 2002). In veterinary medicine, thigh circumference has been used as an objective measurement to document muscle size, and to monitor changes over time (Kramer et al., 2018; and Millis & Levine, 2014).

Limb girth has been considered an indirect method of assessing muscle mass, but previous work has shown it to be a good indicator of actual muscle mass (Millis & Levine, 2014; and Martin et al., 1990). In humans, thigh girth measurements have been shown to be both reliable and reproducible (Doxey, 1987). However, veterinary studies have shown variability and decreased reliability in measuring technique variability between observers (Smith et al., 2013; Bascuñán et al., 2016; and Baker et al., 2010). Several measuring techniques have been reported for measuring limb girth in dogs, however, the utilisation and type of tape measure, different anatomical landmarks, and patient positioning have all been shown to affect measurement reliability (Kramer et al., 2018; Baker et al., 2010; and Smith et al., 2013). A main technique used for measurements of the thoracic limb and pelvic limb involve a tape measure being placed around the thigh or brachium either at the groin or axilla, respectively. However, studies have reported that this method may not be reliable as there is a tendency for a tape measure to slide distally due to the conical shape of the brachium and thigh in dogs (Smith et al., 2013; and Bascuñán et al., 2016). McCarthy et al. (2018) reported a preferred measurement technique for hindlimbs to be at 70% thigh length, from the greater trochanter distally to the lateral fabellae with the measurement reported just proximal to the stifle. However, this requires a series of measurements to determine the location for tape measure placement. Previous studies have reported techniques to measure thoracic limb girth measurements (Tobias et al., 1994; and Barthélémy et al., 2014). Both studies also required a series of measurements to determine the level of the distal third of the humerus for tape measure placement.

Another technique has been suggested that incorporates a consistent landmark within the obtained measurement to increase the reliability of the limb girth measurement (Kramer et al., 2018). Without a specific landmark, there is a wider margin for inconsistency due to the tapering of muscle bellies. Anatomical landmarks such as the greater trochanter of the femur and the acromion process of the scapula have been suggested for the pelvic and thoracic limb measurements, respectively (Kramer et al., 2018). To the authors' knowledge, there are no current studies specifically utilising the greater trochanter and acromion process to assess limb girth measurements in dogs. Additionally, we could not identify any previous studies that compared the girth of the pelvic limb to the thoracic limb.

The purpose of this prospective study was to determine the accuracy of an anatomical landmark incorporated measuring technique and to determine if there is a consistent ratio between pelvic limb (PL) and thoracic limb (TL) measurements in sound dogs. Our goal was to establish a reliable and reproducible reference range for clinical use. If reproducible, this reference range may help clinicians further evaluate dogs during lameness and orthopaedic examinations. Our hypothesis was that the limb girth of sound dogs will have a reproducible PL to TL girth ratio, regardless of weight, body condition score (BCS) or sex.

## Methods

For this prospective study, clients were required to consent to all study parameters and complete a Canine Brief Pain Index (CBPI) prior to patient examination (PennVet, 2023). Initial gait and orthopaedic exams were performed prior to determine if the dog met the study inclusion criteria. Lameness exams were performed at a walk and trot, followed by a standing orthopaedic exam. Physical examinations were performed by a diplomate of the American College of Veterinary Sports Medicine and Rehabilitation (DACVSMR). Physical exams included a Colorado Canine Acute Pain Index Scale (CCAPS) and body condition score (BCS) grading scale out of 9 (Colorado State University, 2006; and Purina Institute, 2023). Patient information including date of birth, body weight, occupation, breed, and sex were recorded. Dogs were required to have an overall CBPI score of 0 out of 10 and show no visible signs of lameness for inclusion. If the inclusion criteria were not met, limb girth measurements were not obtained.

For limb girth measurements, each patient was required to stand square, which was confirmed by both the first observer taking measurements and the second observer recording measurement values. A Gulick II tape measure as used to measure limb girth according to the operator’s manual (Country Technology, Gay Mills, Wisconsin, USA.). Anatomical landmark-incorporated girth measurements were then performed, and all measurements were recorded in centimeters. For the thoracic limbs, the tape measure was placed at the acromion process of the scapulohumeral joint and wrapped around the axillary region as proximal as possible with the tape ending back at the acromion process (Fig. S1, A). A similar technique was used for the pelvic limb measurement, using the greater trochanter as the main landmark and wrapped around the inguinal region as proximal as possible with the tape ending back at the greater trochanter (Fig. S1, B). Three consecutive measurements were made for each limb and the tape measure was completely released from the limb between each measurement. Each limb was measured three times, resulting in a total of 12 measurements per dog.

Average limb girth measurements were calculated from the three trials for all four limbs of each dog. A subsequent average was then made between the right and left thoracic limb (TL) averages to create an overall TL measurement average. The same process was done to create an overall pelvic limb (PL) measurement average. Each patient’s overall TL and overall PL averages were then compared in ratio (PL:TL). This ratio was chosen in this order to have the typically larger anthropometric value as the numerator for easier interpretation.

Simple regression analysis was used to compare the relation between overall pelvic limb and overall thoracic limb measurements using statistical software (IBM, Statistical Product and Service Solutions). Additional variables tested included sex, body weight, and BCS. An independent groups t-test was used to compare PL:TL between males and females. Weight was compared to PL:TL using a simple regression analysis. BCS was compared to PL:TL with a regression analysis. Intraclass correlation coefficient (ICC) was performed utilising Cronbach’s alpha to compare the reliability and continuity of intra-observer variance. Statistical significance was set at P < 0.05.

## Results

A total of 117 dogs were evaluated for this study from June 2019 to January 2020. Two dogs were excluded due to a positive lameness evaluation and a CBPI of greater than 0 in one or more of the categories. Therefore, a total of 115 dogs met inclusion for this study. Of the 115 dogs, 28 were intact males, six were intact females, 37 were spayed females, and 44 were castrated males. Average body weight was 29.8 ± 9.08 (5.2–63.6) kilograms. Average age was 4.4 ± 2.5 (0.5–10.5) years. For BCS, 16 dogs were 4/9, 84 dogs were 5/9, 11 dogs were 6/9, two dogs were 7/9, and two dogs were 8/9. Multiple occupations were reported for the sample of dogs which included 58 companion dogs, 21 explosives detection, 12 narcotics detection, 10 search and rescue, five service, seven recreational sporting dogs, and two show dogs. Breeds included 16 German Shepherds, 26 Labrador Retrievers, 13 Belgian Malinois, 3 Husky, 3 Portuguese Water Dogs, 3 Weimaraners, and 21 mixed breed dogs. Breeds with two or less dogs included Australian Cattle Dog, Australian Shepherd, Beagle, Belgian Tervuren, Border Collie, Boxer, Dalmatian, Dutch Shepherd, Flat-coated Retriever, Goldendoodle, Great Dane, Newfoundland, Pitbull Terrier, Shetland Sheepdog, Spinone Italiano, and standard Schnauzer. The average overall PL:TL ratio of the sample was 1.51 ± 0.05. 52 of 115 dogs had a PL:TL muscle girth ratio of 1.5, corresponding to 3:2. All but nine cases were within two standard deviations of 1.50 (1.40–1.60) (Fig. S2). The correlation between overall PL girth and overall TL girth was strong, with r = 0.97 and r2 = 0.95. A simple regression analysis was used to assess the slope of the relationship of PL girth to TL girth (Fig. S3). The regression line equation was defined as y = 1.956 + 1.448x, where y is predicted overall pelvic limb girth measurement and x is overall thoracic limb girth measurement, or PL = 1.956 + (1.448*TL). Based on statistical analysis, PL:TL was not related to dog weight (P = 0.712), BCS (P = 0.844), or sex (P = 0.247). The intra-class correlation was found to be excellent with an ICC of 0.99. The intraobserver variance was also compared by calculating the standard deviation of the three repeated limb girth measurements for each dog at each limb by the first observer. The overall mean standard deviation of measurements was 0.11 cm ± 0.23 cm.

## Discussion

Limb girth measurement reliability overall has been considered questionable due to intra- and interobserver variation, patient positioning, or the variability in measurement devices used (Kim et al., 2022). To the authors' knowledge, although a landmark-incorporated limb girth measurement has been discussed by Kramer et al. (2018), there are currently no peer-reviewed studies that have evaluated a landmark-incorporated limb measurement for evaluation of muscle mass in dogs. In this prospective study, we performed this anatomical landmark-incorporated girth measurement utilising the greater trochanter of the femur and the acromion process of the scapula. Our results showed that this technique can be reproducible and revealed a ratio between the pelvic limb (PL) and thoracic limb (TL) of 1.5, or 3:2, in the absence of both lameness on visual gait analysis and pain on physical exam.

The main diagnostic for evaluating muscle size in human medicine is considered to be advanced imaging, including both computed tomography (CT) and magnetic resonance imaging (MRI) (Erlandson et al., 2016). Musculoskeletal ultrasound has also been validated to determine muscle size in humans (Mechelli et al., 2019). Further investigation is also required to validate muscle size in dogs using these imaging techniques. Bullen et al. (2017) compared CT to musculoskeletal ultrasound for muscle evaluation in dogs, and only the supraspinatus and infraspinatus were considered valid with ultrasound. Unfortunately, these modalities are limited in veterinary medicine due to financial cost and the need for sedation or general anaesthesia. Therefore, an economical, non-invasive, and reproducible measurement that can be performed in a clinical setting within a short amount of time, like limb girth measurements, would be optimal for veterinary clinicians. However, a recent literature review reported that current techniques for limb girth measurements have been shown to be of questionable reliability (Kim et al., 2022). Previous studies have utilised landmarks, however, anatomical landmarks were used to identify the proximal and distal points to then calculate a percentage of distance at which the actual circumferential limb measurement will be obtained (Smith et al., 2013; Clarke et al., 2020; Barthélémy et al., 2014; Tobias et al., 1994; Monk et al., 2006; Moeller et al., 2010; Baker et al., 2010; McCarthy et al., 2018; and Bascuñán et al., 2016). Measurements requiring a series of calculations prior to obtaining may contribute to variability.

To achieve a reproducible limb girth measurement, a technique with excellent reliability would be required. Our study specifically utilised an anatomical landmark within the measurement, incorporating a palpable portion of bone within the tape measure placement. Our results showed that this technique was reproducible when performed by a single observer. We suspect this type of tape measure placement allows more consistency and prevents tendencies of tape measure sliding that was previously reported due to conical shape of the brachium and thigh (Smith et al., 2013; and Bascuñán et al., 2016).

Sample size may also play an important role in reliability. Previous canine studies evaluating limb girth consist of small sample sizes, ranging from one dog to 35 dogs (Baker et al., 2010; Bascuñán et al., 2016; Moeller et al., 2010; McCarthy et al., 2018; Smith et al., 2013; Clarke et al., 2020; von Pfiel et al., 2020; Barthélémy et al., 2014; and Tobias et al., 1994). Our goal with this study was to evaluate a larger sample size than previous studies, consisting of 115 dogs of predominantly large breeds. A larger sample size resulted in a closer resemblance to a normal distribution curve.

Patient positioning has been reported to contribute to variability in limb girth measurements. Several studies measuring limb girth have been performed in lateral recumbency in both awake, sedated, or anaesthetised dogs (Smith et al., 2013; Clarke et al., 2020; McCarthy et al., 2018; and Monk et al., 2006). Although measured in lateral recumbency, McCarthy et al. (2018) reported measurements in dogs at an estimated standing angle and noted that fully extended limb positions may have more consistency in the lateral recumbent patient, as estimated standing angles in lateral recumbency can result in variability. Previous studies performed in standing position have also shown variability utilising different limb girth measuring techniques (von Pfeil et al., 2020; Bascuñán et al., 2016; Baker et al., 2010; and Moeller et al., 2010). For our study, an awake standing position was preferred for all four limb measurements. Standing position was selected due to practicality and an attempt to find a reproducible measurement technique that did not require the additional risks associated with sedation or general anaesthesia. The authors also preferred a standing position as a weight-bearing stance requires the activation of muscle groups affiliated with locomotion.

Hair clipping was reported to significantly affect limb circumference measurements in one study (Bascuñán et al., 2016), but not statistically significant in another (McCarthy et al., 2018). Our study did not compare hair coat length in dogs, but dogs within the sample predominantly consisted of a short hair coat. However, due to high intra-class correlation, it is possible that hair clipping may not affect results when using the proposed measurement technique in this study. Further investigation is required to determine if this is a significant factor when using a landmark-incorporated limb girth measurement.

Thigh girth comparisons or ratios between contralateral pelvic limbs has remained controversial, as dogs with bilateral joint disease are common (Buote et al., 2009). Gordon-Evans et al. (2011) measured dogs while standing at the proximal quarter of the femur and recorded both the measurement and the ratio of the measurement in comparison to the contralateral pelvic limb at rehabilitation sessions following lateral fabellar suture stabilisation. These measurements were found to be greater in dogs with bilateral cruciate disease than dogs with unilateral disease, but the difference was not statistically significant. The authors of this study (Gordon-Evans et al., 2011) suggested that bilateral muscle atrophy due to bilateral cranial cruciate disease may contribute to a component of greater differences in thigh circumference at 7-weeks post lateral fabellar suture stabilisation. Due to the prevalence of bilateral joint disease in dogs, we elected to compare all four limbs for each dog in our study for sound dogs. As our results showed a consistent PL:TL ratio of 1.5, or 3:2, we believe that this ratio has potential to help pinpoint both unilateral and bilateral musculoskeletal and neurologic diseases.

The authors note that interpretation of a PL:TL ratio may be difficult in the event of muscle atrophy in all four canine limbs. However, in the event of bilateral thoracic limb atrophy, the authors propose that a PL:TL ratio would be less than 1.51 ± 0.05 and subsequently greater than 1.51 ± 0.05 in the event of bilateral PL atrophy. Further investigation is required to confirm this proposal through additional prospective studies. We suspect that unilateral, single limb muscle atrophy requires comparison of all four individual limb measurements for interpretation, as an atrophied limb PL:TL ratio may be greater than or less than 1.51 ± 0.05, while the contralateral PL:TL ratio would be equivocal to 1.51 ± 0.05. Further investigation is required to confirm this proposed theory through additional prospective studies.

Limitations of this study included data collected by a single observer via an unblinded study. The observer in this study was not blinded to the individual value that was measured and recorded. Therefore, knowing previous measurements could potentially lead to bias for subsequent limb measurements. Measurements made by the observer were only occasionally varied which may imply that bias increased the reliability of the measurements to a significant value. However, a previous study performed was not blinded and observers showed poor inter- and intratester reliability for muscle measurements of the brachium, crus, and thigh (Smith et al., 2013). Inter-observer variability was also not evaluated in this study, as there was only one observer obtaining measurements. Further investigation is warranted to perform a blinded study and with additional observers to assess inter-observer reliability and continuity of this landmark-incorporated muscle girth measurement utilised in this study.

The sample selection of dogs utilised in this study needs to be considered as a limitation, as the majority of the dogs were medium to large sized dogs and predominantly breeds with athletic and / or working dog occupations. Although PL:TL ratio was not related to dog weight, BCS, or sex, breed was not statistically evaluated due to lack of diversity in breed selection. Further investigation is warranted to evaluate this measurement technique with dogs of miniature, small, and giant breeds. This investigation should take into consideration comparing the proposed measurement technique in breeds with higher conformations of musculature, such as American Staffordshire Terriers, Cane Corso, and Pit Bull Terriers.

The Gullick II measuring device was previously found to have lower interobserver and intra-observer variation (Baker et al., 2010) and suggested that a spring-loaded tension gauge may also reduce variability. The sole observer was properly trained to use the device and had been using it in practice for multiple years. This study did not test if newly trained observers would be able to replicate these landmark incorporated girth measurements with similar intra-observer reliability. Further investigation is warranted to determine if observers who are newly trained to use a Gullick II tape measure would result in reproducible and consistent PL:TL ratio of 1.5, or 3:2.

In our study, lameness was not assessed by force plate gait analysis or a pressure sensitive walkway. Force plate is considered advanced contextualised care for the evaluation of lameness and therefore peak vertical force and vertical impulse could be utilised to verify clinically sound patients (Brown et al., 2013). In our study, each dog was observed at a walk and trot for subjective, or visual gait analysis by a diplomate of the American College of Veterinary Sports Medicine and Rehabilitation (DACVSMR). However, the sensitivity of objective gait analysis is considered superior to subjective gait analysis (Millis & Ciuperca, 2015; and Quinn et al., 2007). Future studies should consider force plate gait analysis to rule out subtle lameness that might be missed on subjective gait analysis.

The data produced in this study helps establish a sound dog baseline to use as a comparison tool for patients with musculoskeletal disease. We have shown that a landmark-incorporated limb girth measurement in a weight-bearing position can be reproducible. This technique is an inexpensive and noninvasive monitoring tool that may be used by both general practitioners and veterinary specialists for both initial evaluations and further case monitoring. Clinicians utilising a landmark-incorporated limb girth ratio may not only help track case progression, but also help pinpoint subclinical musculoskeletal diseases.

## Supplementary materials

Supplementary material S1 – Figure S1: Illustrated example of a landmark-girth measurement technique for the canine pelvic limb (PL) (A) using the greater trochanter of the femur and for the canine thoracic limb (TL) using the acromion process of the scapula (B).

Supplementary material S2 – Figure S2: Overall sample distribution of the 115 dogs and correlating PL:TL limb girth ratios.

Supplementary material S3 – Figure S3: The average pelvic limb (PL) to thoracic limb (TL) measurements of 115 dogs with a linear regression.

## Acknowledgements

The authors would like to thank Michael Biderman for assistance with statistical analysis. Additionally, the authors would like to give recognition to the Anne Arundel County (MD) Police Department, Mason-Dixon Search Dogs, Amtrak Police Department (Union Station, Washington, DC) K-9s, Johns Hopkins Canine Section, St. Mary’s County (MD) Sheriff’s K9, Starfleet Service Dogs, and the staff at the Carolina Ranch Animal Hospital (Garner, NC) for recruitment of subjects for this study.

## Author contributions

**Elisabeth A. Fox:** Conceptualisation (equal), Methodology (lead), Validation (equal), Formal Analysis, Investigation (supporting), Resources (lead), Data curation, Writing – Original draft, Writing – Review & editing (lead), Visualisation, Project administration. **Kirsty E. Oliver:** Resources (supporting), Writing – Review & editing (supporting). **Matthew W. Brunke: **Conceptualisation (equal), Methodology (supporting), Validation (equal), Investigation (lead), Resources (supporting), Review & editing (supporting), Supervision.

## ORCID

Elisabeth A. Fox: https://orcid.org/0000-0002-1401-1072

Kirsty E. Oliver: https://orcid.org/0000-0001-8611-4524

Matthew W. Brunke: https://orcid.org/0000-0001-5923-0001

## Conflict of Interest

The authors declare no conflicts of interest.

## Disclaimer

Any opinions expressed in articles and other publication types published in Veterinary Evidence are the author's own and do not necessarily reflect the view of the RCVS Knowledge. Veterinary Evidence is a resource to help inform, and the content herein should not override the responsibility of the practitioner. Practitioners should also consider factors such as individual clinical expertise and judgement along with patient’s circumstances and owners’ values. Authors are responsible for the accuracy of the content. While the Editor and Publisher believe that all content herein are in accord with current recommendations and practice at the time of publication, they accept no legal responsibility for any errors or omissions, and make no warranty, express or implied, with respect to material contained within. For further information please refer to our Terms of Use.
